# Weight Loss Patterns and Outcomes Over 12 Months on a Commercial Weight Management Program (CSIRO Total Wellbeing Diet Online): Large-Community Cohort Evaluation Study

**DOI:** 10.2196/65122

**Published:** 2025-01-15

**Authors:** Gilly A Hendrie, Danielle L Baird, Genevieve James-Martin, Emily Brindal, Paige G Brooker

**Affiliations:** 1 Commonwealth Scientific and Industrial Research Organisation Adelaide Australia

**Keywords:** obesity, obesity management, weight loss, internet-based intervention, weight management

## Abstract

**Background:**

A greater understanding of the effectiveness of digital self-management programs and their ability to support longer-term weight loss is needed.

**Objective:**

This study aimed to explore the total weight loss and patterns of weight loss of CSIRO (Commonwealth Scientific and Industrial Research Organisation) Total Wellbeing Diet Online members during their first 12 months of membership and examine the patterns of platform use associated with greater weight loss.

**Methods:**

Participants were Australian adults who joined the program between October 2014 and June 2022 and were classified as longer-term members, meaning they completed at least 12 weeks of the program, had baseline and 12-week weight data, and had a paid membership of ≥1 year (N=24,035). Weight loss and percentage of starting body weight loss were calculated at 3, 6, 9, and 12 months using 3 statistical approaches: (1) multiple imputations method, (2) all available data, and (3) complete data only. Among members with complete data (6602/24,035, 27.5%), patterns of weight loss and gain were examined, and how this related to total weight loss and platform use was explored.

**Results:**

Members were mostly female (19,972/24,035, 83.09%), aged 31 to 50 years (9986/24,035, 41.5%) or 51 to 70 years (12,033/24,035, 50.06%), and most members were classified as overweight or obese (23,050/24,035, 95.9%). Using multiple imputations, the average estimated weight loss was 5.9 (SE 0.0245) kg at 12 weeks, 6.7 (SE 0.0348) kg at 6 months, 6.2 (SE 0.0400) kg at 9 months, and 5.5 (SE 0.0421) kg at 12 months. At 12 months, more than half the members (12,573/24,035, 52.3%) were at least 5% below their starting body weight and 1 in 4 (5865/24,035, 24.4%) were at least 10% below their starting body weight. In the subsample with complete data, the average weight loss at 12 months was 7.8 kg. The most common (961/6602, 14.56% members) weight loss pattern over the first 12 months was 6 months of weight loss, followed by 6 months of weight maintenance. This group had an average weight loss of 10.6 kg at 12 months (11.9% of their starting body weight). In a subgroup of participants who consistently lost weight over the 12-month period (284/6602, 4.3% of the sample), weight loss reached up to 22.3 kg (21.7% of their starting body weight). Weekly platform use was positively associated with total weight loss (*r*=0.287; *P*<.001). Members who used the platform >30 times per week (approximately >4 times/d) were more likely to lose weight in the first 6 months of the program.

**Conclusions:**

This commercial weight loss program was shown to be effective, with 1 in 2 members achieving clinically significant results after 1 year. Greater engagement with the platform was associated with consecutive periods of weight loss and greater weight loss success overall.

## Introduction

### Background

Obesity is a global public health challenge that has substantial individual, societal, and economic impacts. It is predicted that, if the current trends continue, the majority (51%) of the global population will be living with either overweight or obesity by 2035, and 1 in 4 people will be classified as obese [1]. High-income countries tend to have the highest rates of obesity. For example, in Australia, 66% of the adult population is classified as overweight or obese, with approximately 1 in 3 individuals classified as obese [2]. The economic impacts of overweight and obesity are expected to reach 3% of the global Gross Domestic Product by 2035 [1]. The costs to the Australian economy attributable to overweight and obesity were estimated to be Aus $11.8 billion (US $7.4 billion) in 2017 to 2018 [3], and without significant action, these costs may rise to an estimated Aus $87.7 billion (US $54.9 billion) by 2032 [4]. In response to the impacts of obesity, the World Health Organization has set a global target to stop the increases in rates of overweight and obesity, and there have been calls to develop national strategies to address obesity. In line with this, the National Obesity Strategy for Australia aims to “halt the rise and reverse the trend in the prevalence of obesity in adults by 2030” [5]. The strategy acknowledges that achieving this ambitious goal will require coordinated efforts, and if successful, it will significantly enhance the population’s quality of life.

### Commercial Weight Loss Programs

Among the range of approaches to address obesity, improving dietary intake and increasing physical activity are considered useful lifestyle strategies for losing weight. Reviews of commercial weight loss programs have been primarily based on randomized controlled trials and suggest that individuals who complete these programs can achieve significant weight loss in the short term [6]. However, longer-term weight loss, and weight maintenance after weight loss, is challenging [6-8]. The literature, including randomized controlled trials [9] and studies on real-world commercial programs [10,11], commonly reports a pattern of short-term weight loss (at 3 and 6 months) followed by some degree of weight regain by 12 months and beyond.

Longitudinal and longer-term studies need to manage missing data. Randomized controlled trials often use statistical techniques, such as intention-to-treat analysis, to use data from all participants regardless of their adherence or completion of the program. However, a common limitation of real-world type studies that have evaluated the longitudinal effect of weight loss programs is that they often report results from a small proportion of the enrolled participants [12,13]. Therefore, findings are more likely to represent the results of a highly motivated subsample and less likely to represent the expected weight loss for an average participant or be applicable to the broader population. In fact, reported dropout rates for weight loss programs range from 10% to 80% at 12 months [14], often due to reasons associated with lack of, or less than, desirable weight loss [15]. Data from participants included in follow-up analyses would suggest that longer-term weight loss success is a real challenge. Approximately 80% of weight lost is regained within 5 years [16], and only 1 in 5 people are successful in achieving longer-term weight loss [17].

Studies of weight loss maintainers report some common behavioral strategies associated with longer-term success. Frequent self-monitoring of body weight and food intake, high levels of physical activity, and following a low-fat diet were shared behaviors among successful longer-term weight loss maintainers in the National Weight Control Registry [17-19]. Portion control, habitual healthy snack choice, and program engagement have also been shown to be positively associated with weight loss maintenance among individuals enrolled in a digital commercial program [16].

### Purpose of This Study

The Commonwealth Scientific and Industrial Research Organisation (CSIRO) and Digital Wellness have partnered to deliver a commercial, digital version of the CSIRO Total Wellbeing Diet. CSIRO is Australia’s national science agency and Digital Wellness is an Australian digital health solutions company. The CSIRO Total Wellbeing Diet is a weight loss program that has evolved from clinical trials [20-22] to a popular series of books [23] and now a digital platform [24]. The program includes personalized eating plans, customized weekly meal plans, food and exercise diaries, the ability to record and see progress of weight loss, a member forum, and supportive correspondence via email. The CSIRO Total Wellbeing Diet Online program launched in 2014. The results of members who joined the program in the first 5 years have been published previously [24]. In a sample of >60,000 members who joined between 2014 and 2019, the average weight loss of all members was 2.8 kg (3.1% of their starting body weight), and for those who finished the 12-week program, the average weight loss was 4.9 kg (5.3% of their starting body weight). This study focused on longer-term weight loss and therefore used data from members who had joined the program between 2014 and 2022 and who had been members for at least a year. The analysis explored the total weight loss and patterns of weight loss of members during the first 12 months of membership and examined whether the patterns of platform use were associated with greater longer-term weight loss.

## Methods

### Study Design and Participants

Participants of this secondary analysis included all adults who joined the CSIRO Total Wellbeing Diet Online and had data in the platform between October 2014 and June 2022 (n=155,075 people referred to as members). In the registration process, individuals who were aged <18 years or had a BMI that placed them in the underweight category (BMI <18.5 kg/m^2^) were automatically excluded. Participants excluded from this analysis were pseudo-members (ie, platform testers and affiliated staff), individuals whose membership was paid for by their employer because their motivations for weight loss might have been different from those who paid for their own membership, or people living outside of Australia because the context in which they were following the program might be different. Members missing a starting weight as part of the setup process were removed because the primary outcome of weight loss could not be calculated (2172/155,075, 1.4%), and an additional 8 participants were removed because their recorded weight value was deemed invalid or unreliable using cutoff values of <13 kg or >250 kg.

This analysis focused on weight loss among longer-term members, defined as individuals who had completed at least 12 weeks of the program, had weight data recorded at baseline and approximately 12 weeks (61,514/152,895, 40.23%), and maintained a paid membership for about a year or longer. This left a sample of 24,035 longer-term members to examine the longer-term weight loss results of members on the CSIRO Total Wellbeing Diet Online program. To examine the patterns of weight loss over the first 12 months of membership, complete weigh-in data were required for the 4 time points of interest (12 weeks, 6 months, 9 months, and 12 months). There was a subsample 6602 members with complete weigh-in data used for the analysis of weight loss patterns.

### Intervention

A detailed description of the program has been published previously [24]. Briefly, the CSIRO Total Wellbeing Diet Online is a commercial weight loss program managed by Digital Wellness and is available to individuals at a cost of Aus $199 (US $123) for the first 12 weeks and up to Aus $449 (US $278) for 12 months. The diet is a higher-protein, lower–glycemic index dietary pattern, with meal plans consisting of 3 meals and up to 2 snacks each day. The program contains a food group system where portions of food are presented as standard units for each food group. There are 7 food groups: fruit, vegetables, meat and alternatives, breads and cereals, dairy, healthy fats and oils, and indulgences. Meals are designed around a template of standard units, which ensures daily allowances of food groups are met and provides optimal nutrition and energy to promote weight loss. The weight loss program is promoted via media and digital channels, and the Australian public can join the program of their own volition. The user registration process collects information on year of birth, sex, physical activity levels, and weight loss goals to tailor eating plans. A food group unit system allows standard menu templates to be customized by swapping meals using the recipe database.

The platform is a fully responsive web application with an interface that is optimized for viewing on a desktop and mobile devices. The program content provides general information and advice, and weekly tutorials. Self-monitoring and goal setting are key behavior change techniques used within the program. A weight loss goal is provided during the onboarding process. The food diary can be used to log meals and snacks, either by entering prepopulated recipes from the meal plan or by entering individual foods from a comprehensive food database. The food search function allows members to search a database to view information about their composition. The food tracker allows food units and total energy consumed over the day to be logged and reviewed, with graphical feedback provided comparing intake to daily food unit targets. A large database of recipes is available within the platform, and members can swap recipes in and out of the menu plan to suit their taste preferences, time, and cooking skills. Members receive weekly emails on their nominated weigh-in day to remind them to weigh themselves and record their weight into the weight tracker. Progress data are presented in a graph and table form. Members are encouraged to engage with the platform regularly, with recommendations provided to weigh in weekly, use the self-monitoring tools daily, and view program tutorials. The platform also has a forum for members to share their stories or discuss relevant issues with other members. Seasonal content is shared with members, and the program content is reviewed and updated periodically. The platform functionality is updated with a focus on enhancing the user experience and engagement.

### Data Collection and Study Outcomes

Digital Wellness manages the collection and storage of data from the program. Information collected at the time of joining included date of birth; sex; ethnicity; postcode; as well as body measurements such as height, weight, and waist circumference. Other data collected included program details such as paid membership duration and platform use data including entries into the food diary, views of the menu plans, views of exercise plans, views of program content information, forum visits, searches of the food database, and weight entries. These data were provided to the research team in a deidentified format, with each individual member assigned a unique identifier. Weigh-in data were provided for each member weekly from weeks 1 to 13 and then in 3-month blocks thereafter until membership ceased. The last recorded weight was provided within each 3-month block. Platform use data (number of weigh-ins, food diary entries, menu plan views, exercise plan views, program content views, forum views, food search views, and total platform activity) were provided at similar time intervals.

The primary outcome was weight loss, calculated as the difference in kilograms between weight on joining (baseline) and the 4 time points of interest, with a larger number representing greater weight loss. Weight loss was also calculated as a percentage of starting body weight and was categorized into 4 groups: weight gain, weight loss of >0% and <5% of starting body weight, 5% to <10% of starting body weight, and ≥10% of starting body weight lost.

### Ethical Considerations

Ethics approval to conduct this research was received from CSIRO Health and Medical Human Research Ethics Committee (2022_055_LR). As part of registration, participants agreed to their data being shared with CSIRO and used for research purposes; therefore, no direct participant consent was sought. All data were provided to the research team in a deidentified format, and individuals could not be reidentified by the research team. Participants did not receive compensation.

### Statistical Analysis

#### Overview

Statistical analyses were performed using the SPSS statistical software package (version 29; IBM Corp). Descriptive analysis was conducted to describe the characteristics of program members. The average weight loss of members was calculated in three ways: (1) using multiple imputations as a method to replace missing data (24,035/24,035, 100%); (2) using the available weight data (ranged from 18,032/24,035, 75.02% at 3 months to 9413/24,035, 39.16% at 12 months); and (3) using only members with complete data at all time points (6602/24,035, 27.47%).

Weight loss in kilograms and as a percentage of starting body weight were calculated. Weight loss was calculated at each of the time points of interest and by subgroups, including by sex, age group, starting weight status category (normal weight, overweight, or obese [obesity class 1, 2, and 3]), state of residence, quintiles of Socio-Economic Indexes for Areas, and duration of paid membership. Total platform use was aggregated and divided by weeks of membership to estimate an average weekly platform use for each member. The association between platform use and weight loss was assessed using Pearson correlation coefficient, where a positive value indicates a positive association between use and weight loss.

#### Primary Analysis

Weigh-in data were needed to calculate weight loss. The amount of missing weigh-in data increased over time. At 12 weeks, 25% of weigh-in values were missing; at 6 months, 29% were missing; at 9 months, 50% were missing; and at 1 year, 61% were missing. To explore the effect of missing weigh-in data on weight loss estimates, analyses were conducted on the raw data (missing data ignored) before applying the multiple imputations method. The monotone multiple imputation model was used, which included weight loss at each time point, age group, sex, state, starting weight, starting BMI, and Socio-Economic Indexes for Areas quintiles. A total of 20 imputation datasets were created. The model assumed missing data were missing not completely at random. Results presented are the average of separate, identical analyses performed on each of the 20 complete imputation datasets, with appropriate adjustment of SEs to incorporate the added variability from the imputation process.

#### Sensitivity Analysis

Weight loss was also calculated using the available weigh-in data in the system. The amount of weigh-in data ranged from 75.02% (18,032/24,035) at 3 months to 39.16% (9413/24,035) at 12 months. Weight loss was also calculated using only members with complete weight data at all time points (6602/24,035, 27.47%). Weight loss patterns in four 3-month blocks over 12 months were examined and recorded as a sequence. Weight loss during each 3-month period was categorized as weight loss (L), weight gain (G), or weight maintenance (M). Consistent with previous research [25], a change in weight within 1.4 kg of previous weight was considered weight maintenance, and anything greater was considered weight loss or weight gain. For example, if a member lost weight from baseline to week 12 and also lost weight in months 3 to 6, 6 to 9, and 9 to 12, then the weight loss pattern was labelled as L|L|L|L to indicate 4 consecutive periods of weight loss. Alternatively, if a period of weight loss was followed by a period of maintenance and then 6 months of gradual weight gain, the pattern was labelled as L|M|G|G. Total weight loss over 12 months and average weekly platform use were examined by the pattern of weight loss.

## Results

### Characteristics of Members

Longer-term members (24,035/61,514, 39.07%) had an average of 600 days of paid membership (Table 1). The average starting weight was 92.6 kg, and the average BMI was 33.1 kg/m^2^ on commencement of the program. Most members were female (19,972/24,035, 83.09%) and aged between 31 and 50 years (9986/24,035, 41.5%) or 51 to 70 years (12,033/24,035, 50.06%). Nearly all the longer-term members were classified as overweight or obese (23,050/24,035, 95.9%), with 64.68% (15,546/24,035) classified as obese. Approximately one-third of the longer-term members (8291/24,035, 34.49%) were in the highest quintile of socioeconomic status (ie, most advantaged), and similar to the general Australian population, three-quarters of the sample (18,094/24,035, 75.28%) lived in the eastern states of Australia (Victoria, New South Wales, and Queensland).

**Table 1:** Age, starting weight, BMI, and demographic characteristics of members at the start of the program.

		Longer-term members		All members (N=61,514)
		Study sample (n=24,035)	Subsample with complete data (n=6602)	
**Membership duration (d), mean (SD)**		600 (356)	663 (393)	328 (314)
**Starting weight (kg), mean (SD)**		92.6 (19.2)	92.7 (18.4)	91.1 (18.8)
**Starting BMI (kg/m^2^), mean (SD)**		33.1 (6.2)	33.3 (6.0)	32.4 (6.0)
**Age (y), mean (SD)**		52 (11.6)	54 (11.2)	51 (12.4)
**Sex, n (%)**				
	Female	19,972 (83.09)	5593 (84.72)	49,847 (81.03)
	Male	4063 (16.9)	1009 (15.28)	11,667 (18.97)
**Age group (years), n (%)**				
	18-30	1283 (5.34)	218 (3.3)	4711 (7.66)
	31-50	9986 (41.55)	2291 (34.7)	26,232 (42.64)
	51-70	12,033 (50.06)	3823 (57.91)	28,401 (46.17)
	>71	705 (2.93)	263 (3.98)	2118 (3.44)
	Missing	28 (0.12)	7 (0.11)	52 (0.08)
**State, n (%)**				
	New South Wales	7894 (32.84)	2167 (32.82)	19,750 (32.11)
	Victoria	5926 (24.65)	1657 (25.1)	14,998 (24.38)
	Queensland	4274 (17.78)	1133 (17.16)	11,763 (19.12)
	South Australia	2006 (8.35)	567 (8.59)	5161 (8.39)
	Western Australia	1873 (7.79)	508 (7.69)	4849 (7.88)
	Tasmania	566 (2.35)	153 (2.32)	1448 (2.35)
	Northern Territory	263 (1.09)	66 (0.99)	532 (0.86)
	Australian Capital Territory	979 (4.07)	275 (4.17)	2361 (3.84)
	Missing or invalid	254 (1.06)	76 (1.15)	652 (1.06)
**SEIFA^a^**				
	1 (lowest)	2248 (9.35)	604 (9.15)	5890 (9.57)
	2	3492 (14.53)	931 (14.1)	8966 (14.57)
	3	4542 (18.9)	1279 (19.37)	11,808 (19.19)
	4	5203 (21.65)	1490 (22.57)	13,465 (21.89)
	5 (highest)	8291 (34.5)	2220 (33.63)	20,721 (33.69)
**Weight status**				
	Normal weight	957 (3.98)	177 (2.68)	3282 (5.33)
	Overweight	7504 (31.22)	2054 (31.11)	21,046 (34.21)
	Obese	15,546 (64.68)	4366 (66.13)	37,123 (60.35)
	Class 1 obese	8070 (33.58)	2232 (33.81)	20,230 (32.89)
	Class 2 obese	4410 (18.35)	1277 (19.34)	10,310 (16.76)
	Class 3 obese	3066 (12.76)	857 (13.0)	6583 (10.7)
	Missing or invalid	27 (0.11)	5 (0.07)	61 (0.1)

^a^SEIFA: Socio-Economic Indexes for Areas.

Longer-term members with complete data (6602/24,035, 27.47%) were slightly older than the longer-term members generally (54 vs 52 years), with 61.89% (4086/6602) of the members with complete data aged >51 years compared to 53% (12,738/24,035) of all longer-term members. However, their starting weight and BMI were similar, and the proportion classified as obese was also similar (4366/6602, 66.13% vs 15,546/24,035, 64.68%) to longer-term members generally (Table 1).

Compared to all members of the CSIRO Total Wellbeing Diet, longer-term members were, on average, 1.5 kg heavier at the start of the program and had a slightly higher BMI (33.1 vs 32.4 kg/m^2^). The percentage of longer-term members classified as obese was 64.68% (15,546/24,035) compared to 60.35% (37,123/61,514) for all members (Table 1).

### Weight Loss Over 12 Months

Using multiple imputations, the average weight loss of the longer-term members (24,035/24,035, 100%) was estimated as 5.9 kg (or 6.3%) at 12 weeks, 6.7 kg (or 7.2%) at 6 months, 6.2 kg (or 6.6%) at 9 months, and 5.5 kg (or 5.9%) at 12 months. Weight loss for male members was estimated to be 7.0 kg at 12 months compared to 5.2 kg for female members. At 12 months, 57.71% (2345/4063) of male members had lost ≥5% of their starting body weight compared to 51.21% (10,228/19,972) of female members, and 26.43% (1074/4063) of males and 23.99% (4791/19,972) of females had lost 10% of their starting body weight. Overall, it was estimated that 52.31% (12,573/24,035) and 24.4% (5865/24,035) of the members had lost 5% and 10% of their starting body weight, respectively (Table 2).

As a sensitivity analysis, the average weight loss was calculated using the weigh-in data available. The average weight loss of longer-term members was calculated to be 6.4 kg (or 6.8% of starting body weight) at 12 weeks, 7.1 kg (or 7.6%) at 6 months, 7.0 kg (or 7.4%) at 9 months, and 6.3 kg (or 6.7%) at 12 months (Table 3). At 12 months, male members lost an average of 7.7 kg (or 7.1%) and female members lost an average of 6 kg (or 6.6%). At 12 months, just more than half the members (5055/9413, 53.78%) had lost ≥5% of their starting body weight and slightly more than 1 in 4 members (2595/9413, 27.57%) had lost ≥10% of their starting body weight. There were slightly more male members than female members who achieved a weight loss of ≥5% after 12 months (815/1409, 57.84% vs 4250/8004, 53.1%). At 12 months, 29.88% (421/1409) of the male members had lost ≥10% of their starting body weight, which was similar to female members (2174/8004, 27.16%; Table 3).

Weight loss among those members with a valid weight in the system at all time points (6602/24,035, 27.47%) was 6.8 kg (or 7.2%) at 12 weeks, 8.5 kg (or 9%) at 6 months, 8.4 kg (or 8.9%) at 9 months, and 7.8 kg (or 8.2%) at 12 months. Male members in this subgroup lost 9.1 kg (or 8.4%) at 12 months and female members lost 7.5 kg (or 8.2%; Table 3).

Approximately 2 in 3 members (4232/6602, 64.1%) in this subgroup had lost ≥5% of their starting body weight at 12 months, and 1 in 3 members (2342/6602, 35.47%) had lost ≥10% of their starting body weight at 12 months (Table 3).

**Table 2:** Weight loss over 12 weeks at 3, 6, 9, and 12 months calculated using the multiple imputations (MI) method (N=24,035).

Sex, statistic, and time point				Long-term members: MI (no missing; SE)
**Female (n=19,972)**				
	**Weight loss (kg), mean (SE)**			
		12 weeks (n=19,972)	5.5 (0.0247)	
		3-6 months (n=19,972)	6.3 (0.0362)	
		6-9 months (n=19,972)	5.8 (0.0422)	
		9-12 months (n=19,972)	5.2 (0.0449)	
	**Proportion of body weight lost (%), mean (SE)**			
		12 weeks (n=19,972)	6.1 (0.0260)	
		3-6 months (n=19,972)	7.0 (0.0381)	
		6-9 months (n=19,972)	6.4 (0.0447)	
		9-12 months (n=19,972)	5.7 (0.0480)	
	**Proportion losing >5% TBW^a^ (percentage of sample)**			
		12 weeks (n=12,473)	62.45	
		3-6 months (n=12,583)	63.00	
		6-9 months (n=11,224)	56.20	
		9-12 months (n=10,228)	51.21	
	**Proportion losing >10% TBW (percentage of sample)**			
		12 weeks (n=2697)	13.5	
		3-6 months (n=5357)	26.8	
		6-9 months (n=5242)	26.2	
		9-12 months (n=4791)	24.0	
**Male (n=4063)**				
	**Weight loss (kg), mean (SE)**			
		12 weeks (n=4063)	8.0 (0.0703)	
		3-6 months (n=4063)	8.7 (0.0977)	
		6-9 months (n=4063)	7.9 (0.1101)	
		9-12 months (n=4063)	7.0 (0.1133)	
	**Proportion of body weight lost (%),mean (SE)**			
		12 weeks (n=4063)	7.4 (0.0615)	
		3-6 months (n=4063)	8.1 (0.0863)	
		6-9 months (n=4063)	7.3 (0.0979)	
		9-12 months (n=4063)	6.5 (0.1021)	
	**Proportion losing >5% TBW (percentage of sample)**			
		12 weeks (n=2987)	73.52	
		3-6 months (n=2844)	70.00	
		6-9 months (n=2566)	63.15	
		9-12 months (n=2345)	57.72	
	**Proportion losing >10% TBW (percentage of sample)**			
		12 weeks (n=1007)	24.78	
		3-6 months (n=1384)	34.06	
		6-9 months (n=1226)	30.17	
		9-12 months (n=1074)	26.43	
**Total (n=24,035)**				
	**Weight loss (kg), mean (SE)**			
		12 weeks (n=24,035)	5.9 (0.0245)	
		3-6 months (n=24,035)	6.7 (0.0348)	
		6-9 months (n=24,035)	6.2 (0.0400)	
		9-12 months (n=24,035)	5.5 (0.0421)	
	**Proportion of body weight lost (%),mean (SE)**			
		12 weeks (n=24,035)	6.3 (0.0242)	
		3-6 months (n=24,035)	7.2 (0.0349)	
		6-9 months (n=24,035)	6.6 (0.0408)	
		9-12 months (n=24,035)	5.8 (0.0435)	
	**Proportion losing >5% TBW (percentage of sample)**			
		12 weeks (n=15,460)	64.32	
		3-6 months (n=15,427)	64.19	
		6-9 months (n=13,790)	57.37	
		9-12 months (n=12,573)	52.31	
	**Proportion losing >10% TBW (percentage of sample)**			
		12 weeks (n=3704)	15.41	
		3-6 months (n=6741)	28.05	
		6-9 months (n=6468)	26.91	
		9-12 months (n=5865)	24.40	

^a^TBW: total body weight.

**Table 3:** Weight loss over 12 weeks at 3, 6, 9, and 12 months calculated using all available data and for members with complete data (n=6602).

Sex, statistic, and time point				Long-term members (with missing data)							Long-term members with complete data				
				Values, n		Values, mean (SE)		Percentage of sample		Values, n			Values, mean (SE)		Percentage of sample
**Female**															
	**Weight loss (kg)**														
		12 weeks	14,802		5.9 (0.0281)		—^a^		5593			6.4 (0.0454)		—	
		3-6 months	14,332		6.7 (0.0434)		—		5593			8.2 (0.0722)		—	
		6-9 months	10,266		6.7 (0.0622)		—		5593			8.1 (0.0866)		—	
		9-12 months	8004		6.0 (0.0757)		—		5593			7.5 (0.0925)		—	
	**Proportion of body weight lost (%)**														
		12 weeks	14,802		6.6 (0.0287)		—		5593			7.1 (0.0453)		—	
		3-6 months	14,332		7.4 (0.0446)		—		5593			8.9 (0.0721)		—	
		6-9 months	10,266		7.3 (0.0637)		—		5593			8.8 (0.0871)		—	
		9-12 months	8004		6.6 (0.0775)		—		5593			8.2 (0.0935)		—	
	**Proportion losing >5% TBW** ^b^														
		12 weeks	10,123		—		68.5		4139			—		74.1	
		3-6 months	9459		—		66.1		4245			—		76.0	
		6-9 months	6152		—		60.0		3880			—		69.5	
		9-12 months	4240		—		53.0		3546			—		63.5	
	**Proportion losing >10% TBW**														
		12 weeks	2277		—		15.4		1021			—		18.3	
		3-6 months	4234		—		29.5		2278			—		40.8	
		6-9 months	3172		—		30.9		2206			—		39.5	
		9-12 months	2174		—		27.2		1973			—		35.3	
**Male**															
	**Weight loss (kg)**														
		12 weeks	3230		8.4 (0.0795)		—		1009			8.9 (0.1424)		—	
		3-6 months	2827		9.2 (0.1210)		—		1009			10.4 (0.2061)		—	
		6-9 months	1877		8.8 (0.1765)		—		1009			9.9 (0.2363)		—	
		9-12 months	1409		7.7 (0.2045)		—		1009			9.1 (0.2389)		—	
	**Proportion of body weight lost (%)**														
		12 weeks	3230		7.8 (0.0689)		—		1009			8.3 (0.1234)		—	
		3-6 months	2827		8.5 (0.1056)		—		1009			9.6 (0.1788)		—	
		6-9 months	1877		8.1 (0.1535)		—		1009			9.1 (0.2064)		—	
		9-12 months	1409		7.1 (0.1833)		—		1009			8.4 (0.2108)		—	
	**Proportion losing >5% TBW**														
		12 weeks	2477		—		76.8		817			—		81.0	
		3-6 months	2038		—		72.2		788			—		78.3	
		6-9 months	1231		—		65.6		718			—		71.1	
		9-12 months	815		—		57.8		677			—		67.2	
	**Proportion losing >10% TBW**														
		12 weeks	918		—		28.4		330			—		32.7	
		3-6 months	1071		—		37.9		470			—		46.7	
		6-9 months	686		—		36.5		436			—		43.2	
		9-12 months	421		—		29.9		369			—		36.6	
**Total**															
	**Weight loss (kg)**														
		12 weeks	18,032		6.4 (0.0280)		—		6602			6.8 (0.0455)		—	
		3-6 months	17,159		7.1 (0.0420)		—		6602			8.5 (0.0694)		—	
		6-9 months	12,143		7.0 (0.0597)		—		6602			8.4 (0.0821)		—	
		9-12 months	9413		6.3 (0.0716)		—		6602			7.8 (0.0867)		—	
	**Proportion of body weight lost (%)**														
		12 weeks	18,032		6.8 (0.0268)		—		6602			7.2 (0.0431)		—	
		3-6 months	17,159		7.6 (0.0412)		—		6602			9.0 (0.0669)		—	
		6-9 months	12,143		7.4 (0.0589)		—		6602			8.9 (0.0802)		—	
		9-12 months	9413		6.7 (0.0714)		—		6602			8.2 (0.0855)		—	
	**Proportion losing >5% TBW**														
		12 weeks	12,600		—		70.0		4956			—		75.2	
		3-6 months	11,497		—		67.1		5033			—		76.4	
		6-9 months	7383		—		60.8		4598			—		69.8	
		9-12 months	5055		—		53.8		4232			—		64.1	
	**Proportion losing >10% TBW**														
		12 weeks	3195		—		17.7		1351			—		20.5	
		3-6 months	5305		—		30.9		2748			—		41.7	
		6-9 months	3858		—		31.8		2642			—		40.1	
		9-12 months	2595		—		27.6		2342			—		35.5	

^a^Not applicable.

^b^TBW: total body weight.

### Weight Loss Patterns Over 12 Months

Weight loss patterns were examined in 3-month blocks among members with a valid weight in the system at all time points (6602/24,035, 27.47%). The average weight loss of this group was 7.8 kg after 12 months of membership. The most common pattern of weight loss for these members over the first 12 months of membership was weight loss in the first two 3-month blocks (ie, first 6 months), followed by weight maintenance in the second two 3-month blocks of membership (ie, second 6 months). This pattern (L|L|M|M) was observed in 14.6% (961/6602) of the members, and this subgroup achieved an average weight loss of 10.7 kg at 12 weeks and 10.6 kg at 1 year (or 11.9% of their starting body weight; Table 4).

**Table 4:** Platform use (activity per week) and weight loss (kg), described in 3-month blocks over 12 months of membership, by weight loss patterna (n=6602).

Weight loss sequence^b^	Sample, n (%)	Starting body weight (kg)	Platform use (activity per week)					Weight loss (kg)				
			12 weeks	6 months	9 months	12 months	12 weeks		6 months	9 months	12 months	Percentage of body weight (12 months)
L|L|M|M	961 (14.6)	88.9	62.3	37.8	23.7	16.5	7.3		10.7	10.8	10.6	11.9
L|M|M|M	734 (11.1)	84.5	52.9	24.2	17.8	13.1	5.1		5.3	5.0	4.8	5.7
L|L|L|M	590 (8.9)	96.8	64.1	46.9	35.3	24.8	8.7		13.6	16.5	16.6	17.1
L|L|M|G	495 (7.5)	96.2	64.6	37.4	19.1	13.7	8.1		11.6	11.6	8.6	8.9
L|M|G|M	439 (6.6)	89.6	51.2	18.5	12.3	9.7	6.0		6.1	3.3	3.3	3.6
L|M|M|G	366 (5.5)	88.1	56.5	23.0	14.0	11.0	6.3		6.5	6.3	3.6	4.0
L|L|G|M	306 (4.6)	94.0	62.5	31.8	15.1	12.1	7.9		11.0	8.2	8.1	8.5
L|L|L|L	284 (4.3)	102.7	74.7	56.2	45.7	36.3	9.4		15.6	19.5	22.3	21.7
L|L|G|G	270 (4.1)	100.2	66.6	35.0	15.0	10.6	8.9		12.3	9.5	6.3	6.2
L|M|G|G	268 (4.1)	93.4	63.1	20.8	12.3	9.9	7.4		7.5	4.6	1.8	1.8
L|L|L|G	184 (2.8)	101.6	67.8	43.0	31.2	18.6	9.2		14.1	16.8	13.9	13.6
L|G|M|M	183 (2.8)	87.6	49.4	14.3	11.7	9.4	5.5		2.9	2.8	2.7	3.1
L|G|G|M	137 (2.1)	95.3	52.0	15.2	11.3	10.1	6.3		3.7	0.8	0.7	0.7
L|L|M|L	129 (2.0)	96.7	54.8	34.4	24.3	22.7	7.9		11.8	11.9	14.3	14.8

^a^Data are presented for weight loss sequence groups containing ≥2% of the total sample.

^b^Weight loss during each 3-month period was categorized as weight loss (L), weight gain (G), or weight maintenance (M). Consistent with previous research [25], a change in weight within 1.4 kg of previous weight was considered weight maintenance, and anything greater was considered weight loss or weight gain.

The second most common pattern was weight loss recorded in the first 3-month block and then maintenance for the remainder of the year. This group (L|M|M|M, 734/6602, 11.1% of this sample) lost an average of 5.1 kg in 12 weeks (5.7% of their starting body weight) and maintained this loss through to 1 year. The other common pattern was weight loss over the first three 3-month blocks, followed by weight maintenance (L|L|L|M, 590/6602, 8.94% of this sample). This group was heavier when they started the program than the more common L|L|M|M and L|M|M|M groups and lost 16.6 kg over 12 months, equivalent to 17.1% of their starting body weight. Although less common, those achieving weight loss in each 3-month block (L|L|L|L, 284/6602, 4.3% of this sample) were among the heaviest members at the start of the program, and they lost an average of 22.3 kg over 12 months, which was equivalent to 21.7% of their starting body weight (Table 4).

### Platform Use by Weight Loss Pattern

**Figure 1:**
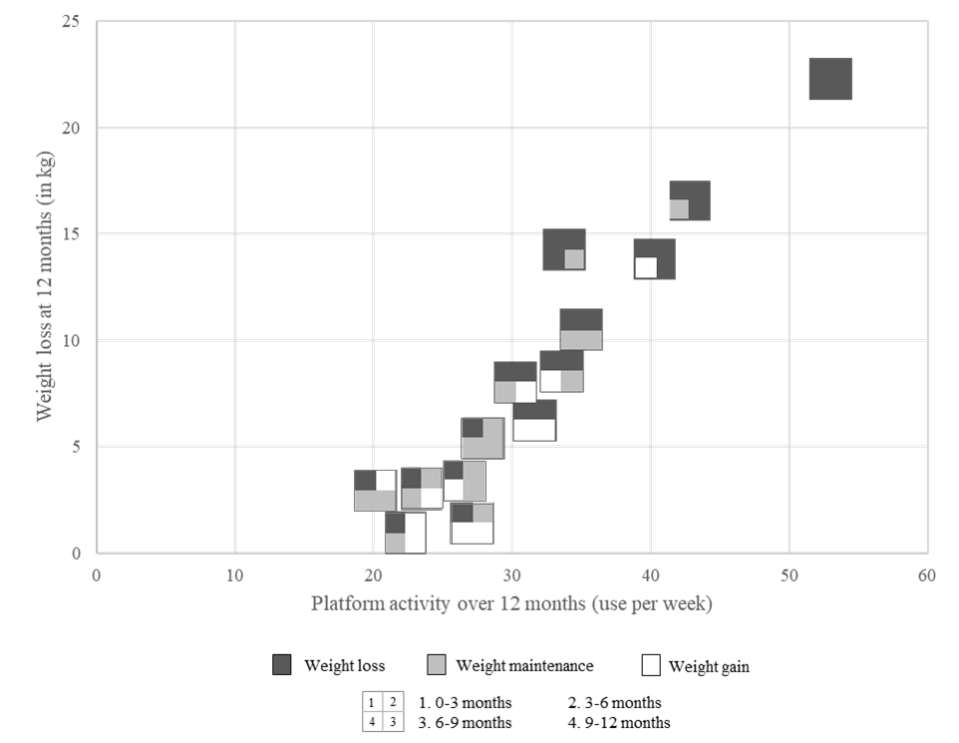
Platform use and total weight loss over 12 months for the most common patterns of weight loss (n=6602).

There was a positive relationship between the average weekly platform use and weight loss over the first 12 months of membership, with higher weekly platform use associated with greater weight loss (*r*=0.287; *P*<.001). Table 3 shows the relationship between platform use, weight loss, and the pattern of weight loss. For example, weekly platform use in the group that consistently lost weight (L|L|L|L) was 24% to 56% higher at each time point throughout the year than other weight loss patterns where use and weight loss were lower. Figure 1 shows the platform use and weight loss at 12 months for each weight loss pattern. The quadrants of the box represent 3-month intervals, with darker colors indicating weight loss and lighter colors indicating weight gain. It was evident that when platform use exceeded ≥30 times per week approximately >4 times per day), the pattern of weight loss included loss for the first 6 months of the program, whereas the groups using the platform for <30 times week recorded a loss in the first 3 months, followed by a period of maintenance or gain (Figure 1). It is also evident that within clusters of weight loss patterns, for example, losing weight in 3 of the 4 blocks of time over 12 months, total weight loss was higher when use was higher (Figure 1).

## Discussion

### Principal Findings

This study examined the longer-term weight loss of members of a commercial weight loss program, the CSIRO Total Wellbeing Diet Online. In a large sample of 24,035 members who had approximately 12 months of paid membership, the average estimated weight loss was 5.9 kg at 12 weeks, 6.7 kg at 6 months, 6.2 kg at 9 months, and 5.5 kg at 12 months. At 12 months, it was estimated that more than half the members (12,573/24,035, 52.3%) remained at least 5% below their starting body weight and 1 in 4 (5865/24,035, 24.4%) were at least 10% below their starting body weight. A modest reduction in weight of 5% has been accepted as clinically meaningful [26], and 5% to 10% is a recommended goal for medically supervised weight loss, as outlined in guidelines for managing overweight and obesity [27,28]. On a smaller subset of members with complete weigh-in data at 3, 6, 9, and 12 months, 64.1% (4232/6602) and 35.5% (2342/6602) reached the recommendations of 5% and 10% body weight loss, respectively. The most common weight loss pattern on this program was 6 months of weight loss, followed by a 6-month period of weight maintenance, with this group achieving a total of 10.6 kg weight loss over 12 months. Although less common, when weight loss was achieved in each of the four 3-month blocks of time, the average weight loss over 12 months was 22.3 kg. Achieving higher weight loss was associated with higher platform use, specifically at a frequency of approximately ≥4 times per day.

### Comparison to Other Literature

The health benefits of modest weight loss are well established. A 5% body weight loss is associated with improved systolic and diastolic blood pressure, glucose control, and cholesterol [29]. However, achieving this level of weight loss is difficult. A cohort study of >18 million health care–seeking Americans found that 1 in 10 adults achieved a 5% body weight loss [30]. Among successful individuals, approximately half report to do it on their own and the other half through a formal program [31]. The do-it-yourself approach can work, and when in a controlled trial (which likely overrepresents effectiveness), up to 1 in 4 people were reported to achieve 5% body weight loss after 12 months [32]. Commercial weight loss programs are an effective option for weight loss, and digital programs, in particular, might be more accessible. The weight loss results of this program compare favorably to similar commercial or digital weight loss programs. For example, an evaluation of another commercial weight loss program, *WW* (formerly known as *Weight Watchers*), reported an average weight loss of 3.8 kg at 3 months and 4.4 kg at 12 months, with 42.8% of participants achieving a loss of 5% body weight at 12 months [32], compared to 52.3% (12,573/24,035) for the CSIRO Total Wellbeing Diet Online. This is one of the few examples of published weight loss results from a commercial weight loss program with a longer follow-up, although the sample size is smaller than the one presented here. Another example of an evaluation of a commercial program is a cross-country comparison of *Noom Coach*, which included 18,459 participants and reported weight losses across countries ranging from 3% to 3.7% of starting body weight loss at 16 weeks, with 27% to 33% (cross-country range) achieving 5% body weight loss [33]. Meanwhile, *MetaWell*, a digital weight loss program, reported comparable short-term (ie, 3 months) weight loss results to the CSIRO Total Wellbeing Diet Online (5.6 vs 5.9 kg for CSIRO Total Wellbeing Diet) in a larger sample of approximately 250,000 members [34]; however, no longer-term follow-up data have been reported yet. Key strengths of this study are the large sample from a commercial weight loss program, with longer-term follow-up and the use of multiple imputations to account for missing data in the estimation of weight loss. Without the use of statistical methods to account for missing data, which can be significant for self-recorded weight data in commercial platforms, the weight loss results are likely to be more favorable and, therefore, less generalizable in terms of expected weight loss outcomes.

The results of this study are consistent with other studies where a weight loss “tick” curve is commonly reported, meaning that initial weight loss success is followed by a plateau at or approximately 6 months and then some weight regain by 12 months [9]. Although the average weight loss difference at 6 and 12 months on this program was 1.2 kg, this would be considered weight maintenance according to the standard definition [25]. Different weight loss patterns are rarely explored beyond the short term [35]. In this study, weight loss patterns were examined in 3-month blocks of time over 12 months. The 3 most common patterns, accounting for one-third of members in this subgroup analysis, involved various combinations of weight loss and weight maintenance, without periods of weight gain. These findings contrast with another study on weight loss patterns from an internet-based weight management program [36], which involved daily weighing over a 1-year period. It found a shift from weight loss directly to weight gain, with weight regain starting much earlier, approximately 11 weeks, and not preceded by a weight maintenance period [36]. Half the sample in this study achieved weight loss in the first 6 months, and most often, this was followed by additional weight loss or maintenance. When weight loss was recorded for ≥3 periods throughout 12 months, the average weight loss was approximately 15 kg regardless of whether the other was a period of maintenance, loss, or gain. This is an important message for the public when embarking on a weight loss journey. Periods of weight gain and maintenance are common, and the trajectory of a longer-term journey will likely not be linear; however, meaningful overall weight loss can still be achieved with time.

There is compelling evidence that increased self-monitoring is associated with greater success in weight management [37]. How to best promote and sustain self-monitoring, as well as the necessary dose required, remain areas of investigation. A previous weight loss study that tracked self-monitoring in a digital system showed that there were no significant differences in time spent self-monitoring based on amount of weight loss at 6 months; however, there were differences in the number of times people logged in. Those who lost >5% of their starting body weight logged into the system approximately 2.5 times per day compared to 1.5 times per day for those who lost <5%, and log-ins were 2.7 times versus 1.7 times per day for those losing >10% compared to <10% of their starting body weight [38]. This supports the results of this study, where those with the highest platform use had a pattern of sustained weight loss and a greater total weight loss over 12 months. These users were interacting with the platform >30 times a week (or >4 times per day), which is higher, but given that the CSIRO Total Wellbeing Diet Online system allows for weight, diet, and exercise monitoring, it would be expected that daily interaction is more regular.

There are several challenges with sustained engagement with digital programs, notably attrition [39]. These are made more significant by growing evidence that sustained engagement may be more important than self-monitoring alone. In a study comparing degrees of weight loss [40], highly successful people were those who lost more weight initially and recorded continued weight loss over time, while less successful people lost smaller amounts of weight initially and then regained half that weight back over time. In the initial 6-month period of that study, there were no differences in the frequency of self-monitoring for moderately and highly successful people; however, longer-term self-monitoring remained high for those in the most successful group, recording significantly more self-monitoring entries than other groups. Together with our observations, this suggests that sustained self-monitoring may be more important for longer-term weight loss results. Self-monitoring in digital programs is typically measured by interactions with the system, which can also serve as a proxy for motivation. A small study in women showed a sustained increase in autonomous motivation at 5% weight loss but a decline for failing to reach this milestone [41]. It also suggested that motivation at week 4 predicted adherence to self-monitoring and weight loss. Thus, achieving clinically significant weight loss may increase motivation, which then improves self-monitoring.

It is clear that digital interventions need to leverage self-monitoring to support more successful weight management. System interactions are one indication of a person’s motivation, emotions, and actions, and it is likely that greater appreciation of these personal factors will be needed to address use attrition. Supporting this, a recent review of 497 self-management randomized controlled trials for obesity found that information sharing, self-monitoring, and goal setting were frequently used techniques in interventions, but a broader scope is needed in interventions to consider social support, emotional management, and shared decision-making in obesity self-management interventions, which are all important outcomes for patients [42]. Interventions for obesity will also need to evolve to responsibly incorporate artificial intelligence to enhance the user experience and support more people in their weight loss journey. In behavioral interventions, artificial intelligence could analyze patterns in datasets to predict behavioral and clinical outcomes early, allowing for more personalized content that enhances learning and motivation [43]. This approach could potentially improve weight loss outcomes for people living with obesity. The use of artificial intelligence might also enhance the user experience throughout the varying stages of weight management [44]. Early weight loss success provides motivation for continued efforts and later success, but a greater understanding of the “ups and downs,” or patterns, of longer-term weight loss is needed.

### Strengths and Limitations

Published data on the longer-term weight loss results from real-world commercial programs are limited. A strength of this study is the large sample of members used to report weight loss results, which is significantly larger than those published for other commercial programs. Weight loss was calculated in several ways, including the use of multiple imputation to address missing data. Missing data can be problematic in real-world programs, and using only the available data may inflate weight loss results, as these data could represent more motivated individuals who use the platform features more often. Multiple imputations as an approach helps to reduce bias and accounts for some of the uncertainty in imputed values by creating multiple complete datasets, which are separately analyzed and combined. A total of 20 datasets were created with values imputed using several different demographic variables known to be associated with weight loss, such as age, sex, and starting weight, among others. As a result, the weight loss results presented are more likely to be reflective of what might be achieved by a member who joins the program in their own everyday context and not a selected subsample [16]. Another strength of this study was the examination of weight loss patterns over an extended period. This analysis was conducted in a subsample of members with complete data, but the sample was still large enough to examine weight loss patterns in four 3-month blocks over 12 months. To the authors’ knowledge, no other commercial program has examined weight loss in this way. These results provide a deeper understanding of the cycles of weight loss success and are critical to managing the expectations of those embarking on a weight loss journey. The CSIRO Total Wellbeing Diet is delivered through a partnership between Australia’s national science agency and a digital health company (Digital Wellness). This is a unique partnership in which both organizations are dedicated to enhancing engagement with the platform to improve weight loss success and retention in the program, offering mutual public health and commercial benefits.

There are some limitations of this study that should be considered. While the sample was large, a limitation was that most members were female, and individuals from the lowest socioeconomic groups were underrepresented compared to the general Australian population. The cost associated with joining the program might be a barrier for those with lower income. Exploring strategies to attract a diverse member base that is more representative of the broader Australian population is a focus of ongoing research. Although real-world trials provide insight into the “true” success of people who follow a program of their own volition, a second limitation is that real-world, commercial programs are limited in what data they can collect. The registration process for this program collects various demographic data, but other factors, such as past dieting history or motivation, were not collected. Such information may allow for better predictions of weight loss success. A third limitation is the measurement of engagement used in this study. Platform use provided an indication of engagement with the program, but more objective measures of compliance, such as dietary intake, may better represent program compliance.

### Conclusions

This analysis presents one of the most comprehensive evaluations of a commercial weight loss program, providing insights on weight loss success over 1 year among a large cohort of members as well as examining patterns of weight loss in a smaller subsample to better understand the “ups and downs” in achieving longer-term weight loss success. Given the prevalence of obesity and the challenges in losing weight, more evidence-based programs that achieve meaningful weight loss are needed. These results show that the CSIRO Total Wellbeing Diet Online is effective for longer-term weight loss with approximately 1 in 2 members achieving clinically significant results after 1 year. Members who engaged with the platform more were able to achieve multiple periods of weight loss over the year, and this was associated with greater weight loss success overall. Future research should focus on understanding how to drive motivation for early and sustained engagement, knowing that self-monitoring is an important factor influencing this complex health challenge. In practice, the findings on the patterns of weight loss within a longer-term journey should be used to help prepare people for a realistic weight loss journey.

## Abbreviations

CSIRO: Commonwealth Scientific and Industrial Research Organisation

## Data Availability

The datasets generated and analyzed during this study are not publicly available because they are considered commercial-in-confidence but are available from the corresponding author on reasonable request.
