# Correction: Weight Loss Patterns and Outcomes Over 12 Months on a Commercial Weight Management Program (CSIRO Total Wellbeing Diet Online): Large-Community Cohort Evaluation Study

**DOI:** 10.2196/71665

**Published:** 2025-02-05

**Authors:** Gilly A Hendrie, Danielle L Baird, Genevieve James-Martin, Emily Brindal, Paige G Brooker

**Affiliations:** 1 Commonwealth Scientific and Industrial Research Organisation Adelaide Australia

In “Weight Loss Patterns and Outcomes Over 12 Months on a Commercial Weight Management Program (CSIRO Total Wellbeing Diet Online): Large-Community Cohort Evaluation Study” ([JMIR 2025;27(1):e65122) the authors noted several errors:

In the Methods section of the Abstract, the sentence:

Among members with complete data (6602/24,035, 27.5%), patterns of weight loss and gain were examined, and how this related to total weight loss was explored.

Has been revised to:

Among members with complete data (6602/24,035, 27.5%), patterns of weight loss and gain were examined, and how this related to total weight loss and platform use was explored.

In the Methods section, the following sentence:

This analysis focused on weight loss among longer-term members, defined as individuals who had completed at least 12 weeks of the program, had weight data recorded at baseline and approximately 12 weeks (61,514/152,895, 1.4%), and maintained a paid membership for about a year or longer.

Has been revised to:

This analysis focused on weight loss among longer-term members, defined as individuals who had completed at least 12 weeks of the program, had weight data recorded at baseline and approximately 12 weeks (61,514/152,895, 40.23%), and maintained a paid membership for about a year or longer.

Finally, in Table 2, n values have been added for the categories under the "Sex, statistic, and time point" heading. The row header "Female" has been revised to: "Female (n=19,972); the row header "Male" has been revised to: "Male (n=4063); and the row header "Total" has been revised to: "Total (n=24,035)".

The correction will appear in the online version of the paper on the JMIR Publications website on February 5, 2025, together with the publication of this correction notice. Because this was made after submission to full-text repositories, the corrected article has also been resubmitted tothose repositories.

